# Whole-Genome Sequence of a European Clone II and OXA-72-Producing *Acinetobacter baumannii* Strain from Serbia

**DOI:** 10.1128/genomeA.01390-15

**Published:** 2015-12-10

**Authors:** Laurent Dortet, Rémy A. Bonnin, Delphine Girlich, Dilek Imanci, Sandrine Bernabeu, Nicolas Fortineau, Thierry Naas

**Affiliations:** a Bacteriology-Hygiene Unit, Assistance Publique-Hôpitaux de Paris, Bicêtre Hospital, Le Kremlin-Bicêtre, France; b Associated French National Reference Center for Antibiotic Resistance: Carbapenemase-Producing *Enterobacteriaceae*, Le Kremlin-Bicêtre, France; c EA7391 “Structure, dynamic, function and expression of broad spectrum β-lactamases,” Université Paris Sud, Université Paris-Saclay, Faculte de Medecine, Le Kremlin-Bicêtre, France; d Molecular Genetics and Hormonology Department, Assistance Publique-Hôpitaux de Paris, Bicêtre Hospital, Le Kremlin-Bicêtre, France

## Abstract

We report here the draft genome sequence of a carbapenem-resistant *Acinetobacter baumannii* strain isolated from a patient, a strain which previously stayed in Serbia. This isolate possessed the *bla*_OXA-72_ carbapenemase gene. The draft genome sequence consists of a total length of 3.91 Mbp, with an average G+C content of 38.8%.

## GENOME ANNOUNCEMENT

*Acinetobacter baumannii* is a Gram-negative opportunistic pathogen responsible for nosocomial infections mainly in immunocompromised patients. Its propensity to acquire resistance genes and survive in the hospital environment leads to outbreaks in health care facilities and mainly in intensive care units (ICU). *A. baumannii* is responsible for diverse infections, such as bacteremia, urinary tract infections, and skin and wound infections (1).

The emergence of carbapenem resistance in *A. baumannii* is of great concern, since these agents are often the last resort for treating *Acinetobacter* infections. In this species, the main carbapenem resistance mechanism is the production of a carbapenem-hydrolyzing Ambler class D β-lactamase (CHDL) (2). The main acquired CHDL belong to the OXA-23, OXA-24/40, OXA-58, OXA-143, and OXA-235 groups (2). *A. baumannii* also contains a gene encoding a naturally occurring CHDL, the *bla*_OXA-51_-like gene, which may be overexpressed by insertion sequences providing strong promoter sequences.

Whereas *bla*_OXA-23_ genes are widespread on all continents, the *bla*_OXA-72_ gene, a variant of the *bla*_OXA-40_ gene, is restricted to three main regions: South America, in Brazil and Colombia (3, 4); southern Asia, in Japan and Taiwan (5, 6); and Eastern Europe, mainly in Croatia and one description in Lithuania (7, 8).

Genomic DNA was extracted using the UltraClean microbial DNA isolation kit (Mo Bio Laboratories) from overnight cultures in LB agar (Bio-Rad, Marnes-la-Coquette, France). The quantification of genomic DNA was performed using a Qubit fluorometer (Life Technologies, Carlsbad, CA) and then adjusted at 0.2 ng/µl for the library preparation. The Nextera XT DNA sample preparation kit (Illumina, San Diego, CA) was used for the preparation of a DNA library. Next, the library was sequenced on an Illumina MiSeq 2000 sequencer with v3 chemistry using 2 × 75-bp paired-end reads.

Sequencing resulted in 2,611,036 reads, with an average length of 75.17 bp. From the raw data, 2,593,454 reads were mapped on the 206 contigs assembled using CLC Workbench version 8.5. The average length of the 206 contigs was 19,003 bp, and the total length was 3,914,647 bp. The G+C content was in accordance with *A. baumannii* species, at 38.8%. These contigs were further annotated using the RAST server (http://rast.nmpdr.org/), which predicted 3,710 coding sequences (CDSs) in the genome.

These coding sequences include subsystems involved in essential metabolism of the bacteria, including cell wall (116 CDSs), RNA (177 CDSs), protein (219 CDSs), and DNA (89 CDSs) metabolism. Accessory features were also present, such as those conferring resistance to antibiotics and toxic compounds, e.g., β-lactamases, arsenic, and copper.

*In silico* analysis using the CGE server (https://cge.cbs.dtu.dk/) revealed that this isolate belonged to the European clone II, a widespread clone associated with carbapenemase dissemination. We describe here the full-genome sequence of the first OXA-72-producing *A. baumannii* strain from Serbia.

### Nucleotide sequence accession numbers.

This whole-genome shotgun project has been deposited at DDBJ/EMBL/GenBank under the accession no. LKIB00000000. The version described in this paper is version LKIB01000000.
